# “Guidelines Recommendations on the Treatment of Tricuspid Regurgitation. Where Are We and Where Do We Go With Transcatheter Valve Intervention”

**DOI:** 10.3389/fcvm.2018.00037

**Published:** 2018-04-12

**Authors:** Alec Vahanian, Eric Brochet, Jean-Michel Juliard

**Affiliations:** Department of Cardiology, Bichat hospital, University Paris VII, Paris, France

**Keywords:** tricuspid regurgitation, transcatheter valve intervention, TTVI, guidelines, tricuspid surgery, TAVI

## Abstract

Tricuspid regurgitation (TR) is an important clinical problem because it is frequent and carries a poor prognosis when it is left uncorrected. However, there is still a lack of awareness of tricuspid disease in the medical community. The indications for evaluation and surgical interventions in patients with TR were recently updated in the ESC/EACTS guidelines. Transcatheter tricuspid valve intervention (TTVI), almost exclusively valve repair, is at an early stage of development as only a few hundreds of patients have been treated. The first-in-man valve implantation was very recently performed. The recent ESC/EACTS Guidelines state that “The potential role of transcatheter tricuspid valve treatment in high-risk patients needs to be determined”. We shall review here which lessons of interest for TTVI can be learned from the Guidelines as regards evaluation and indications for surgery and try to imagine what could be the place of TTVI in the Guidelines in the future.

## Introduction

Tricuspid regurgitation (TR) is an important clinical problem because it is frequent and carries a poor prognosis when it is left uncorrected (1–7). However there is still a lack of awareness of tricuspid disease in the medical community (8).

The indications for evaluation and surgical interventions in patients with TR were recently updated in the ESC/EACTS guidelines (9). Transcatheter tricuspid valve intervention (TTVI), almost exclusively valve repair, is at an early stage of development as only a few hundreds patients have been treated (10, 11). The first-in-man valve implantation were very recently performed (12). This explains the statement in the ESC/EACTS Guidelines: “The potential role of transcatheter tricuspid valve treatment in high-risk patients needs to be determined”.

We shall review here which lessons of interest for TTVI can be learnt from the Guidelines as regards evaluation and indications for surgery and try to imagine what could be the place of TTVI in the Guidelines in the future.

## General Comments

The recent ESC/EACTS guidelines (9) stress the importance of the awareness of tricuspid disease especially in patients with significant mitral valve disease. The patients with severe TR, especially secondary TR, are often at an advanced stage of the disease and require a comprehensive cardiac and non - cardiac evaluation (13). The patient selection is more difficult here than for mitral regurgitation or aortic stenosis and requires expertise. As a consequence the indication for TTVI should be taken in Valve Centres, by a multidisciplinary Heart Team in order to optimize the therapeutic decision (9).

 The management strategy, as advocated in the Guidelines, should follow a systematic stepwise approach trying to answer the following questions: Is the regurgitation severe? What is the mechanism of the regurgitation? Is the patient symptomatic? Are there contra-indications to any intervention on the valve? Is surgery contraindicated or high risk? Is a transcatheter intervention feasible? What is the decision of the Heart Team?

## Imaging

Imaging plays a key role. Most of the statements made in the Guidelines will apply to surgery as well as TTVI.

 Echocardiography is the main examination. It assesses the “Carpentier’s triade” i.e., valve anatomy, lesions and dysfunction, separating primary from the most frequent secondary TR.

It also evaluates the severity of the regurgitation using an integrative approach combining semi-quantitative and quantitative evaluation (9, 14). A new scale for severity, adding “massive and torrential” degrees, has been recently proposed but the additional prognostic value of these new grades remains to be proven (15).

 More attention should be paid to the evaluation of right ventricular function (RV) using tridimensional imaging by echocardiography or cardiac magnetic resonance (CMR). CMR is considered the reference method even if precise thresholds for reversibility of RV dysfunction remain largely unknown in the field of valvular disease (9, 16, 17).

CT, which is not necessary before tricuspid valve surgery, will play a key role in the TTVI era by assessing the characteristics of the tricuspid annulus and the feasibility of each specific transcatheter techniques, in particular the relationship with adjacent structures such as the right coronary artery (18).

 Right heart catheterisation should be performed when needed to evaluate the pulmonary vascular pressures/resistances because of the limitations of echocardiography in such cases.

 Finally imaging during TTVI remains a challenge and the choice of one single method, or more likely multimodality imaging, should be based on new evidence (18).

### Indications for Intervention

Several lessons from surgical experience should be kept in mind when considering TTVI.

As regards the choice of the technique (10), based on the surgical experience (19), the Guidelines state that “Valve repair is preferable to valve replacement. Ring annuloplasty, preferably with prosthetic rings, is key to surgery for secondary tricuspid regurgitation Valve replacement should be considered in primary TR or when the tricuspid valve leaflets are significantly tethered in secondary TR and the annulus is severely dilated”.

These statements may explain why the current hemodynamic results of TTVI are suboptimal when using the first generation devices in patients with unfavourable anatomy such as very severe annular dilatation. In addition the devices currently available, such as annuloplasty or spacer, cannot treat primary TR when they are used in isolation. The feasibility of Mitraclip in degenerative TR has been documented in only a few case reports (20). In addition this technique cannot be used in patients with TR of rheumatic origin or post endocarditis. Thus awaiting more experience using combination of specific TTVI repair techniques, valve replacement may appear to be the most promising technique in primary TR. Finally the potential use of heterotopic valve implantation has to be further evaluated in the end stage disease.

The indications for TTVI in secondary TR could be expected to be as follows, with all the necessary reservations at this early stage of development.

 In the near future intervention may only be considered in inoperable/high-risk patients with severe TR. Patients with severe TR after a previous mitral intervention represent the most frequent scenario. In such cases intervention should be considered early but only when it is not “futile”. Severe pulmonary hypertension (21), severe LV or RV dysfunction, or significant residual left-sided valve disease should be eliminated before considering any intervention on the tricuspid valve. When RV dysfunction is “extreme”, it is unlikely that any intervention on the tricuspid valve will change the prognosis (22). The guidelines did not define any precise thresholds as regards RV function for contraindicating intervention because evidence is lacking. The decision here should individualized and made by the Heart team. It is likely that the indications for TTVI might be a bit more “permissive” than those of surgery if the goal is to reduce symptoms and/or usage of drugs at an acceptable risk. In the other patients the decision will be between medical therapy or heart transplantation.

 The recent ESC/EACTS guidelines insisted on the importance of an early treatment of TR, when surgery is performed only on the tricuspid valve or in combination with the surgical correction of a mitral valve disease. Mitral and tricuspid transcatheter techniques could potentially be combined in patients in the same way as in surgery. If combined transcatheter intervention is doable and affordable, TTVI should ideally be performed during the same session or early after the mitral procedure. Recent short series have suggested that it is feasible with good results (23).

 In a more distant future, if both tricuspid and, even more so mitral, transcatheter intervention prove to be effective and durable in patients at lower risk, the combination of the two techniques will be even more desirable.

In patients undergoing mitral valve surgery, the Guidelines recommend tricuspid surgery in selected cases even if the degree of TR is less than severe based on the degree of annulus dilatation or progressive deterioration of RV function. These early indications cannot apply to TTVI in the near future.

 Finally, the ideal approach of secondary TR caused by tricuspid annular dilatation related to chronic atrial fibrillation is largely unexplored and the decision between the specific treatment of atrial fibrillation or tricuspid surgery or THV should be based on new evidence.

## Conclusions

The role of transcatheter tricuspid intervention is likely to increase in the future based on several factors: increased awareness of tricuspid disease – improvement of technology - search for evidence. If these prerequisites are fulfilled TTVI will help transcatheter valve treatment on any valve to move towards the surgical standards.

## Author Contributions

AV was involved in the whole process of drafting and reviewing the Perspective, EB and J-MJ contributed with their expertise in Imaging and Surgery.

## Conflict of Interest Statement

VA declares that he had been paid for talks by consultancies Abbot vascular, Edwards Life Sciences, Mitraltech and there was no commercial or financial relationships involved with regards to this article leading to any conflict of interest.

The other authors declare that the research was conducted in the absence of any commercial or financial relationships that could be construed as a potential conflict of interest.

The reviewer DR and handling Editor declared their shared affiliation.

## References

[1] Arsalan M Walther T Smith RL Grayburn PA Taramasso M Vanermen H Tricuspid regurgitation diagnosis and treatment. Eur Heart J (2017) 38(9):634–8. 10.1093/eurheartj/ehv487 26358570

[2] Taramasso M Vanermen H Maisano F Guidotti A La Canna G Alfieri O The growing clinical importance of secondary tricuspid regurgitation. J Am Coll Cardiol (2012) 59(8):703–10. 10.1016/j.jacc.2011.09.069 22340261

[3] Nath J Foster E Heidenreich PA Impact of tricuspid regurgitation on long-term survival. J Am Coll Cardiol (2004) 43(3):405–9. 10.1016/j.jacc.2003.09.036 15013122

[4] Topilsky Y Nkomo VT Vatury O Michelena HI Letourneau T Suri RM Clinical outcome of isolated tricuspid regurgitation. JACC Cardiovasc Imaging (2014) 7(12):1185–94. 10.1016/j.jcmg.2014.07.018 25440592

[5] Dreyfus GD Martin RP Chan KM Dulguerov F Alexandrescu C Functional tricuspid regurgitation: a need to revise our understanding. J Am Coll Cardiol (2015) 65(21):2331–6. 10.1016/j.jacc.2015.04.011 26022823

[6] Vassileva CM Shabosky J Boley T Markwell S Hazelrigg S Tricuspid valve surgery: the past 10 years from the Nationwide Inpatient Sample (NIS) database. J. Thorac. Cardiovasc. Surg. (2012) 143(5):1043–9. 10.1016/j.jtcvs.2011.07.004 21872283

[7] Puls M Lubos E Boekstegers P von Bardeleben RS Ouarrak T Butter C One-year outcomes and predictors of mortality after MitraClip therapy in contemporary clinical practice: results from the German transcatheter mitral valve interventions registry. Eur Heart J (2016) 37(8):703–12. 10.1093/eurheartj/ehv627 26614824 PMC4761401

[8] Iung B Delgado V Lazure P Murray S Sirnes PA Rosenhek R Educational needs and application of guidelines in the management of patients with mitral regurgitation. A European mixed-methods study. Eur Heart J (2018). 10.1093/eurheartj/ehx763 29300869

[9] Baumgartner H Falk V Bax JJ De Bonis M Hamm C Holm PJ 2017 ESC/EACTS Guidelines for the management of valvular heart disease. Eur Heart J (2017) 38(36):2739–91. 10.1093/eurheartj/ehx391 28886619

[10] Rodés-Cabau J Hahn RT Latib A Laule M Lauten A Maisano F Transcatheter therapies for treating tricuspid regurgitation. J Am Coll Cardiol (2016) 67(15):1829–45. 10.1016/j.jacc.2016.01.063 27081024

[11] Latib A Mangieri A Transcatheter tricuspid valve repair: new valve, new opportunities, new challenges. J Am Coll Cardiol (2017) 69(14):1807–10. 10.1016/j.jacc.2017.02.016 28385309

[12] Navia JL Kapadia S Elgharably H Harb SC Krishnaswamy A Unai S First-in-human implantations of the navigate bioprosthesis in a severely dilated tricuspid annulus and in a failed tricuspid annuloplasty ring. Circulation (2017) 10(12):pii: e005840 10.1161/CIRCINTERVENTIONS.117.005840 29246915

[13] Taramasso M Hahn RT Alessandrini H Latib A Attinger-Toller A Braun D The international multicenter TriValve registry. JACC: Cardiovascular Interventions (2017) 10(19):1982–90. 10.1016/j.jcin.2017.08.011 28982563

[14] Lancellotti P Tribouilloy C Hagendorff A Popescu BA Edvardsen T Pierard LA Recommendations for the echocardiographic assessment of native valvular regurgitation: an executive summary from the European Association of Cardiovascular Imaging. Eur Heart J Cardiovasc Imaging (2013) 14(7):611–44. 10.1093/ehjci/jet105 23733442

[15] Hahn RT Zamorano JL The need for a new tricuspid regurgitation grading scheme. Eur Heart J Cardiovasc Imaging (2017) 18(12):1342–3. 10.1093/ehjci/jex139 28977455

[16] Baumgartner H Bonhoeffer P De Groot NM de Haan F Deanfield JE Galie N ESC Guidelines for the management of grown-up congenital heart disease (new version 2010). Eur Heart J (2010) 31(23):2915–57. 10.1093/eurheartj/ehq249 20801927

[17] Saremi F Hassani C Millan-Nunez V Sánchez-Quintana D Imaging evaluation of tricuspid valve: analysis of morphology and function with CT and MRI. AJR Am J Roentgenol (2015) 204(5):W531–42. 10.2214/AJR.14.13551 25905959

[18] van Rosendael PJ Delgado V Bax JJ The tricuspid valve and the right heart: anatomical, pathological and imaging specifications. EuroIntervention (2015) 11(Suppl W):W123–7. 10.4244/EIJV11SWA35 26384177

[19] Navia JL Nowicki ER Blackstone EH Brozzi NA Nento DE Atik FA Surgical management of secondary tricuspid valve regurgitation: annulus, commissure, or leaflet procedure? J Thorac Cardiovasc Surg (2010) 139(6):1473–82. 10.1016/j.jtcvs.2010.02.046 20394950

[20] Fam NP Connelly KA Hammerstingl C Ong G Wassef AW Ross HJ Transcatheter Tricuspid Repair With MitraClip for Severe Primary Tricuspid Regurgitation. J Invasive Cardiol (2016) 28(12):E223–4. 27922813

[21] Galiè N Humbert M Vachiery JL Gibbs S Lang I Torbicki A 2015 ESC/ERS Guidelines for the diagnosis and treatment of pulmonary hypertension: The Joint Task Force for the Diagnosis and Treatment of Pulmonary Hypertension of the European Society of Cardiology (ESC) and the European Respiratory Society (ERS): Endorsed by: Association for European Paediatric and Congenital Cardiology (AEPC), International Society for Heart and Lung Transplantation (ISHLT). Eur Heart J (2016) 37(1):67–119. 10.1093/eurheartj/ehv317 26320113

[22] Kammerlander AA Marzluf BA Graf A Bachmann A Kocher A Bonderman D Right ventricular dysfunction, but not tricuspid regurgitation, is associated with outcome late after left heart valve procedure. J Am Coll Cardiol (2014) 64(24):2633–42. 10.1016/j.jacc.2014.09.062 25524343

[23] Nickenig G Kowalski M Hausleiter J Braun D Schofer J Yzeiraj E Transcatheter treatment of severe tricuspid regurgitation with the edge-to-edge mitraclip technique. Circulation (2017) 135(19):1802–14. 10.1161/CIRCULATIONAHA.116.024848 28336788

